# Antioxidant/Pro-Oxidant Actions of Polyphenols From Grapevine and Wine By-Products-Base for Complementary Therapy in Ischemic Heart Diseases

**DOI:** 10.3389/fcvm.2021.750508

**Published:** 2021-11-03

**Authors:** Veronica Sanda Chedea, Liliana Lucia Tomoiagǎ, Ştefan Octavian Macovei, Dan Claudiu Mǎgureanu, Maria Lucia Iliescu, Ioana Corina Bocsan, Anca Dana Buzoianu, Crinuţa Maria Voşloban, Raluca Maria Pop

**Affiliations:** ^1^Research Station for Viticulture and Enology Blaj (SCDVV Blaj), Blaj, Romania; ^2^“Iuliu Hatieganu” University of Medicine and Pharmacy, Cluj-Napoca, Romania; ^3^Department of Pharmacology, Toxicology and Clinical Pharmacology, “Iuliu Hatieganu” University of Medicine and Pharmacy, Cluj-Napoca, Romania

**Keywords:** antioxidant/pro-oxidant activity, grape pomace, grape seed, polyphenols, resveratrol, ischemic heart diseases

## Abstract

Grape pomace and grape seeds, by-products of the wine industry, and grapevine cane resulting from grapevine pruning are cheap matrices containing important amounts of polyphenols. While there is a continuous need of introducing new ways of these by-products valorization, we propose their use as a source of bioactive polyphenols for complementary therapy in ischemic heart diseases. As oxidative stress plays an important role in these diseases, by their antioxidant/pro-oxidant properties, these compounds, mainly flavan-3-ols, procyanidins, and resveratrol may counteract the damage of the oxidative stress. For instance, to some extent, the grape seed extract, considered as an antioxidant nutritive supplement, may have pro-oxidant activity as well, depending on dose, duration of administration, and other dietary components. *In vitro* studies confirm that the antioxidant activity of this extract might be mediated by pro-oxidant *o*-quinones and oxidation products of the polyphenols from grape and winery byproducts, indicating that quinones, as oxidation products, are involved in the modulation of the antioxidant/pro-oxidant balance at the cellular level in the case of catechin-type compounds, in the absence or presence of oxidative stress inducers. *In vivo*, studies indicate that a grape pomace-rich diet results in a significant increase of the total antioxidant status in the plasma, liver, spleen, and kidneys. Also, the administration of grape pomace shows antioxidant activity with positive effects on health. In this context, the present review aims to present the most recent research focused on the antioxidant/pro-oxidant actions of the bioactive polyphenols from grapevine and wine byproducts, in conditions of ischemic heart diseases as assessed *in vitro* or *in vivo*.

## Introduction

Generally, the food chain including the wine industry generates besides the final product, byproducts that contain a substantial proportion of nutrients originating from the input materials (1). According to the United Nations Food and Agriculture Organization (FAO) fruits and vegetable waste production are considered the highest as compared with all types of foods, reaching up to 60%. The high amounts of resulted by-products, their impact on the environment as well as their nutritional composition, make these matrices a significant topic for careful recovery and valorization (1) in the framework of the circular model of economy. This concept represents also a great opportunity, not only to reduce greenhouse gas emissions and fossil fuel (2) but also to contribute to the generation of multiple valuable ideas on how to valorize the waste into different value-added by-products, transforming it into raw material for other industries and applications (cosmetics, pharmaceuticals, food industry, animal feedstock, and others).

One of these high potential fruits suitable for by-products valorization is the grape (*Vitis vinifera*). Currently, grapes are the most cultivated around the world, with 77.1 million tons in 2019 (3), from which 57% was for wine grape production, 36% for table grape utilization, and 7% for dried grapes (4, 5). *Vitis vinifera* is a key growing system characterized by underutilized woody and fiber-rich biomass by-products that can be valorized by reducing the carbon footprint of its high-quality products (grapes and wine). A by-product of pruning and summer trimming, grapevine cane which usually is destroyed by burning, can also be regarded as a widely available potential source of natural resveratrol (RESV) (4, 5). The wine processing industry produces high amounts of by-products like grape pomace (GP) that accounts for 13–25% of grapes total weight. Grape pomace is of particular interest because of its rich chemical composition, especially phenolic compounds (6). Overall, all grape by-products were reported to contain high quantities of phenolics like phenolic acids, flavonols, flavones, flavanones, tannins, flavan-3-ols, proanthocyanidins and, anthocyanins, and stilbenes (7–9). These compounds have been reported to possess multiple pharmacological activities like anticancer (10), antifungal (11), antibacterial (12), antioxidant (8), and anti-inflammatory activities (10). One of the most studied is the antioxidant capacity because of phenolics can act as free radical scavengers, hydrogen donators, metal chelators, and singlet oxygen quenchers (13). However, it was also reported that phenolic compounds can act as pro-oxidants if conditions favorable for autoxidation are present (e.g., high pH, high transition metal ions concentrations, the presence of oxygen molecules) (14). Thus, depending on the environment, some phenolic compounds, especially small molecules, can act either as antioxidants (electron donors) either as pro-oxidants (electron acceptors) (14). Dual antioxidant and pro-oxidant activities were already reported in the literature for a wide range of phenolic compounds like phenolic acids (e.g., gallic, protocatechuic, syringic, vanillic, ellagic, caffeic, coumaric, chlorogenic, and ferulic acids), flavonoids (e.g., myricetin, quercetin, rutin, and kaempferol), flavan-3-ols (e.g., catechin and epicatechin) and anthocyanidins (e.g., delphinidin and malvidin) (14, 15).

Cardiovascular diseases (CVDs) are the leading cause of global morbidity and mortality. In 2019 CVDs accounted for an estimated 17.9 million mortalities worldwide, representing 32% of global deaths (WHO). People living in low- and middle-income countries are more exposed to pre-mature deaths of CVD origin. Among CVDs, ischemic heart disease (IHD) is the most prevalent (16). Ischemic heart disease is a pathological process that is characterized by an imbalance between the demand and supply of myocardial oxygen as a consequence of the reduced cardiac blood flow (17). The causes can be multiple from atherosclerotic obstruction, microvascular dysfunction, coronary artery vasospasm, or congenital anomalies. Ischemic heart disease is often used as coronary artery disease (CAD), being considered synonyms (17, 18). Thus, IHD or CAD is considered a multifactorial phenomenon influenced by the non-modifiable (e.g., genetics, gender, age, family history) or by modifiable risk factors (e.g., obesity, lipid profile, smoking, alcohol, low fruit and vegetable intake, physical inactivity or other psychosocial variables) (19). Current treatments are aiming to reduce the addressable risk factors, especially atherosclerotic disease progression by using classical treatments and/or by counseling the patients on dietary and lifestyle changes. The classical treatments include several classes of compounds. β-blockers are used for the reduction of myocardial oxygen demand and to decrease heart rate and contractility; calcium channel blockers to reduce the systemic vascular resistance translated in a decrease myocardial contractility; nitrates to induce venous dilation influencing heart oxygen demand by a decrease pre-load and demand, renin-angiotensin-aldosterone blockers by the reduction of blood pressure and infarct size and antiplatelet drugs to prevent the formation of thrombus in the coronary arteries. Additionally, lipid-lowering drugs are also used to reduce the risk of heart attacks (20, 21).

Despite the complex management of IHD that is already focused on primary prevention, improved diagnosis, and treatment, globally it remains a major public concern producing enormous health and economic burdens (22). Therefore, key strategies targeted at the prevention of IHD or CVD are expected from all sectors to have global significance with a positive impact. To best accomplish this, a collaborative effort could be performed among the wine industry, medical and pharmaceutical research to valorize grapevine and wine byproducts rich in polyphenols. This can be possible due to the strong link between polyphenols and CVD, and their major impact on oxidative stress, one of the principal pathophysiological processes involved in the pathogenesis of IHD. Thus, polyphenols from grapevine and wine industry by-products can target several oxidation markers derived from excessive interactions with ROS, RNS, and RCS like oxidation of DNA (oxidized purine and pyrimidine bases like 8-OH-dG), or specific proteins (protein carbonyls, oxidized aminoacids), lipids (MDA, isoprostanes, ox-LDL and their antibody) and carbohydrates (23, 24).

Hence, this work aims to offer a complex overview of the antioxidant/pro-oxidant activity of grapevine and wine by-products polyphenols referring to the *in vivo* and *in vitro* studies in the perspective of IHD prevention.

## Grapevine and Wine By-Products—Valuable Sources of Polyphenols

**Figure 1:**
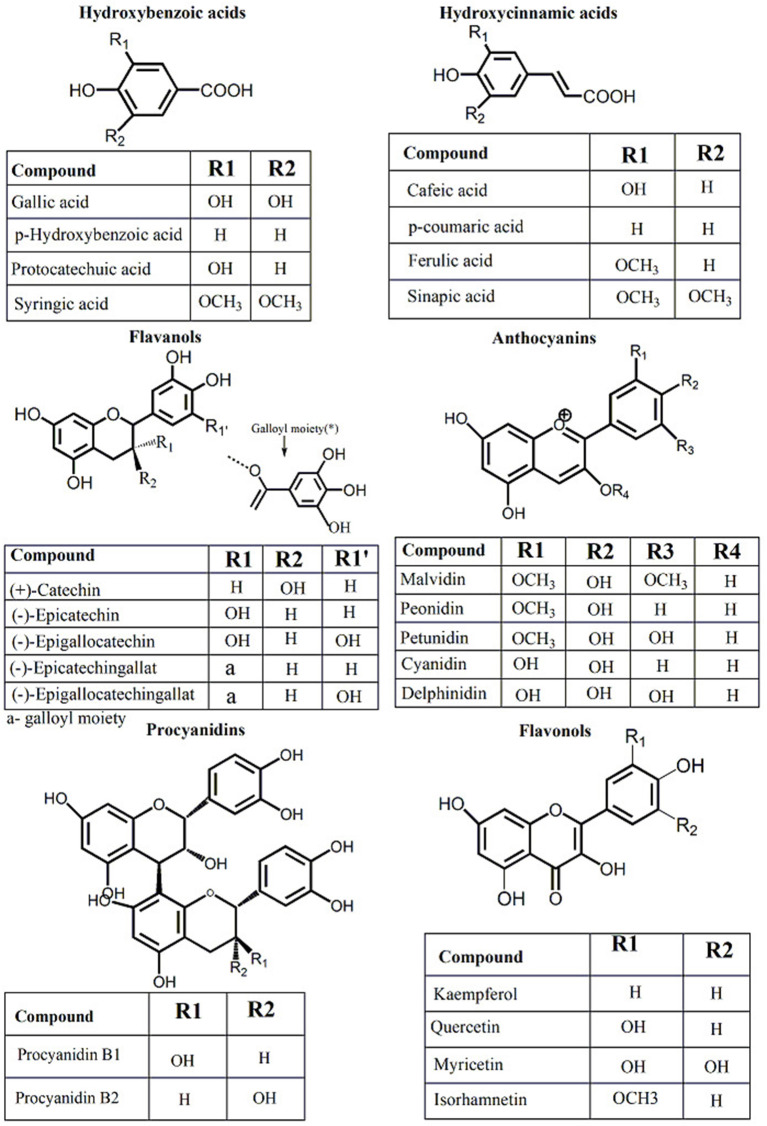
Chemical composition of principal phenolic compounds identified in grapevine and wine by-products.

The production of wine from *Vitis* ssp. grapes is carried out in different steps: grape collection, destemming, crushing, and pressing (25). From the process of winemaking, two different by-products mainly accumulate grape stems and grape pomace (GP) (skins, seeds, and lees) (25). Grape pomace is frequently destined for distillation and production of alcoholic beverages, like grappa in Italy and tsikoudia and tsipouro in Greece, but there is no real utilization of stems except for composting (25). Grape pomace represents ~25% of the grape's weight and, besides distillation most of GP is reused in the vineyards in the carbon cycle (26). Thus, attention should be directed toward other sustainable practices to optimize GP valorization. Among the alternatives used in GP valorization special interest was observed in the food and pharmaceutical industries. The use of GP and grape seeds (GS) in functional foods (dietary fiber + polyphenols), food industry (biosurfactants), pharmaceutical (food supplements like grape pomace and grape seed powder), and cosmetics (grapeseed oil + antioxidants) (26). Finding alternatives for GP utilization is more and more motivated by the increased consumer demand toward natural compounds and their awareness toward alternative uses of waste and by-products. Also, GP, and GS are very rich sources of polyphenols (up to 8%) (27), and they already have a grounded tradition in food and cosmetic sectors as natural additives (coloring agents, conservative agents, antioxidants, or nutritional additives) (28). Polyphenols from GP and GS are mainly represented by monomeric flavan-3-ols like catechin, epicatechin, and gallocatechin (Figure 1) (29). Procyanidin dimers, trimers, highly polymerized procyanidins, and phenolic acid (gallic acid) precursors are also present (Figure 1) (27, 30, 31). Among monomers, catechin and epicatechin are the representative flavanols found in GS and grape skin, having similar levels, or higher epicatechin levels according to grape varieties, geographical area, climate, growing or harvesting conditions (32, 33). Procyanidin B1 was found representative in the grape skin (34). Other compounds like C4–C8 procyanidin dimers were predominantly reported in GS, with procyanidin B2 in higher concentrations (35). Anthocyanins are an important class of polyphenols present in red grapes but not in white ones. Delphinidin, cyanidin, petunidin, peonidin, and malvidin are the most important anthocyanins found in red grapes, and they differ in the B ring substitution (Figure 1). The mono-glucoside forms of delphinidin, cyanidin, petunidin, peonidin, and malvidin are the most common substitutions (36). Compared to *Vitis vinifera* L. grapes, other grape species, such as *Vitis labrusca* and American grape species, have been reported to possess both, 3-mono-glucoside and 3,5-di-glucosides anthocyanin forms (37). As in the case of the other polyphenol classes, GP resulted from the red winemaking, contains a large part of these antioxidant compounds originating from the input material, the grapes (34, 38). In addition to polyphenols, GP contains important quantities of dietary fiber (46/100 g), carbohydrates (29/100 g), protein (8/100 g), and lipids (8/100 g) (39).

**Figure 2:**
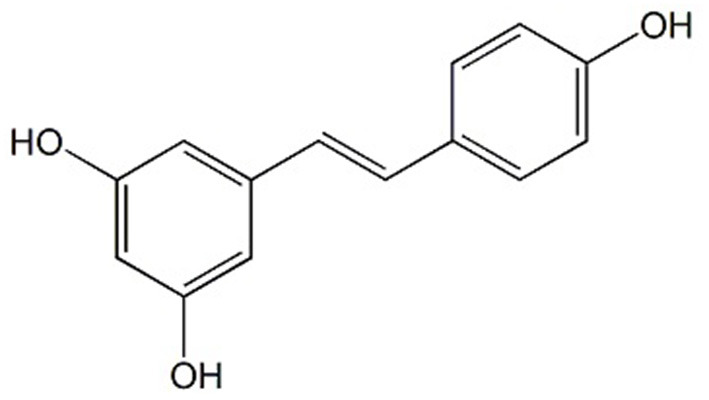
Chemical structure of Resveratrol.

Another phenolic compound found in wine and grapevine tissues and their by-products, with significant benefits in human health, is resveratrol (3,5,4′-trihydroxy-stilbene, RESV) (Figure 2). RESV is produced in the epidermis of leaves and the skin of grapes, particularly when the grapevine is infected with *Botrytis cinerea* (40). In wine, the RESV concentration varies according to wine type: in red wines can be even higher than 580 μg per 100 mL while in white wines, it is much lower (~68 μg per 100 mL) (41). This discrepancy can be explained by the fact that for obtaining the white wine, the skin is removed sooner in the winemaking process, resulting in less RESV being extracted. In addition, red wine has a higher concentration of trans-resveratrol than white wine (29). The latter, on the other hand, contains a greater concentration of cis-resveratrol, which is very sensitive to light (42). RESV and its derivatives levels in plants are influenced by a variety of variables including environmental conditions, abiotic (light, UV radiation), and biotic stress (*Botrytis cinerea* and *Plasmopara viticola* infection) (43). Even though RESV is found in over 72 species of plants (44) some of the main dietary sources are wine, grapes, and grape products as well as their by-products (29, 45).

## Polyphenols Significance in Cardiovascular Diseases

It is already known that the development of CVD is influenced by several factors. Changes in cholesterol and lipid metabolism, the inflammation and oxidative processes characteristic at the vascular wall level, endothelium cellular disruption and intra-lumenal platelet activation and aggregation are the most common pathophysiological processes characteristics for CVD diseases. Some of these processes like atherosclerotic lesions or endothelial dysfunction can be observed in the earlier stages of disease development and therefore are targeted in disease prevention (46). According to literature data, several candidates biomarkers were linked to pathophysiological changes in CAD. For example, the prognosis in stable CAD was associated with myocardial stress and remodeling biomarkers like B-type natriuretic peptide (BNP), N-terminal pro-B-type natriuretic peptide (NT-proBNP), Mid-regional pro-A-type natriuretic peptide (MR-proANP), Mid-regional pro-adrenomedullin (MR-proADM), growth differentiation factor 15 (GDF-15), soluble suppression of tumorigenicity 2 (sST2), Renin, Copeptin and Pro-endothelin-1 (47). Further, the myocardial injury was correlated with cardiac troponins (troponin I and troponin T). Inflammatory processes were linked with C-reactive protein (CRP), Pentraxin 3, Growth differentiation factor-15, Soluble suppression of tumorigenicity (sST2), Myeloperoxidase, Osteoprotegerin, Lipoprotein-associated phospholipase A2, Interleukin-6 and others (47). On the other hand, the acute coronary syndrome was strongly linked to malondialdehyde low-density lipoprotein (MDA-LDL) as a sensitive biomarker of plaque destabilization (46) and with myeloperoxidase (MPO) that, in high concentrations, also contribute to plaque destabilization. Choline and Whole blood choline (WBCHO) were also found in high concentrations after activation of ischemia (46), alongside the increased circulating levels of free fatty acids and ischemia modified Albumin (46). More accessible, an altered profile of serum lipid level is considered one of the most important risk factors for CAD (48). Thus, the reduction of LDL cholesterol that is directly linked to atherosclerosis development and can predict high CVD risks can be considered an easy and important therapeutic target in reducing the CVD risks (48).

Overall, multiple known markers are used to predict CAD or CVDs but considering that their morbidity prevalence is still in the forehead, earlier intervention in the identification and treatment of associated CVD risk factors is needed. Hence, continuous efforts are put to discover new preventive therapies useful in the fight of reducing CVD risks factors. In this sense, collective evidence indicates that polyphenols possess multiple pharmacological effects with positive effects against CVD factors (49–52).

**Figure 3:**
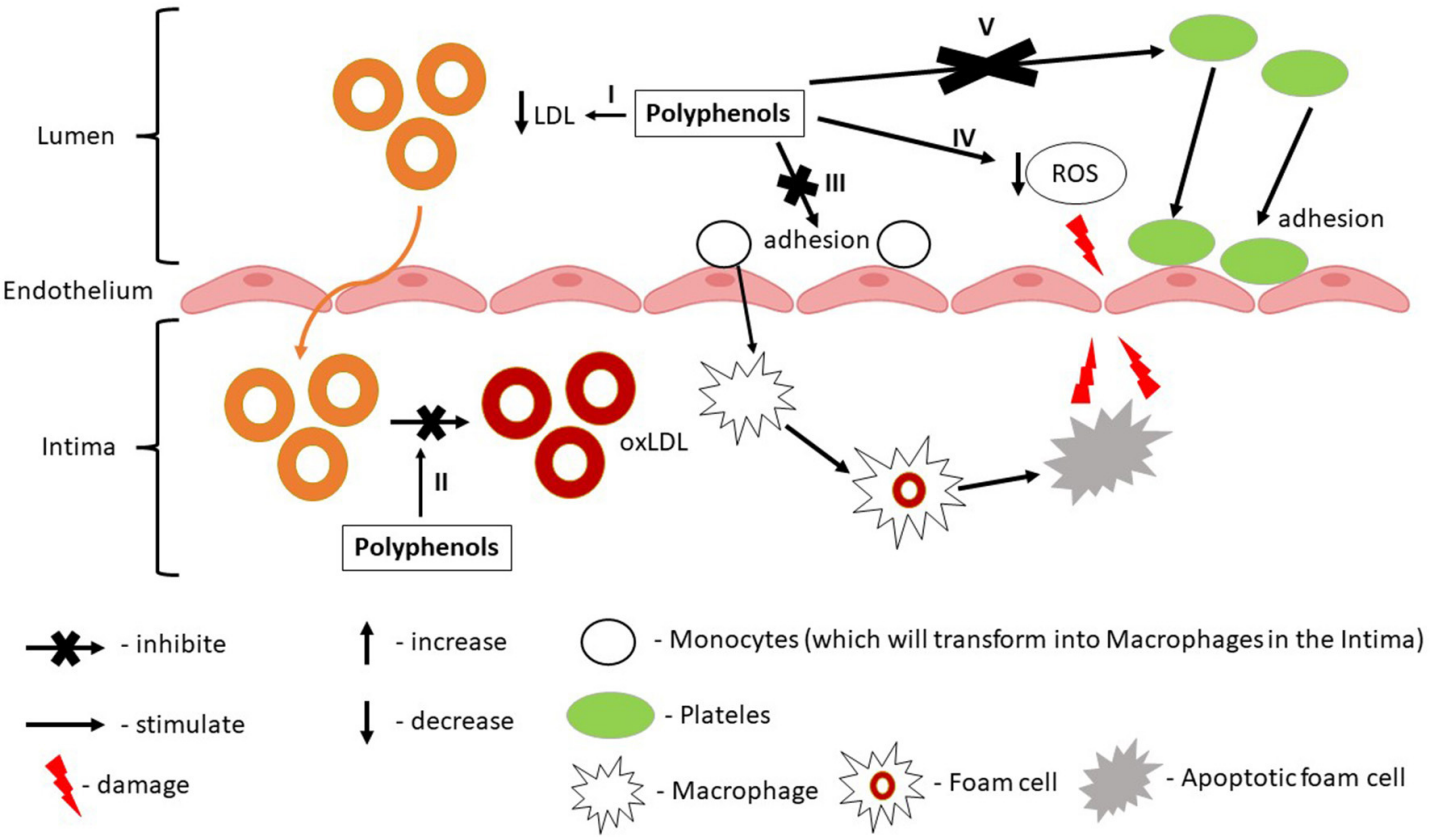
Synergistic and prophylactic effect of grapevine and wine by-products polyphenols in ischemic heart disease. I—Polyphenols reduce LDL levels; II—Polyphenols reduce oxLDL formation; III—Polyphenols reduce monocytes adhesion by reducing the expression of VCAM-1; IV—Polyphenols reduce ROS production; V—Polyphenols inhibit platelets activation and adhesion by reducing the expression of PECAM-1.

Many overlapping mechanisms characteristic for CVD, particularly IHD, were found to be influenced by polyphenols. Of these mechanisms, polyphenols were found to reduce myocardial oxygen consumption and/or to increase oxygen supply (8, 53), to enhance myocardium metabolism following ischaemia/reperfusion (53, 54), to inhibit platelet aggregation (51, 55), thrombosis (55), to regulate lipid metabolism (52), to reduce inflammation (56), to inhibit atheromatous plaques formation (50), to promote endothelial cell repair (57), to protect remaining myocardial cells or to restore myocardial contraction (58). According to these mechanisms, polyphenols will act on multiple molecular targets involving different synergistic protective pathways. For example, referring to polyphenols role in atherosclerotic lesions or endothelial dysfunction (significant in early IHD prevention) it was observed that they have the ability to reduce atherosclerotic progression (59). The proposed mechanisms are related to the improvement of lipid profile (increased HDL, reduced LDL, and total cholesterol). Also, polyphenols can inhibit NADPH oxidase complex, NO release, endothelial activation or can reduce monocyte adhesion to the endothelium (59). Alongside, polyphenols can reduce the production of chemokines, pro-inflammatory cytokines, and angiogenic factors. They were also shown to modulate enzymatic signaling associated with T-cell proliferation, B-cell activation, and cytokine production (59). Polyphenols can modulate endothelial function through the regulation of endothelial cell adhesion molecules expression. In addition, polyphenols can reduce the expression of some adhesion molecules like vascular cell adhesion molecule 1 (VCAM-1), intercellular adhesion molecule 1 (ICAM-1), platelet, and endothelial cell adhesion molecule 1 (PECAM-1) known for their contribution in the early plaque development (57). More than that, polyphenols radical-scavenging properties have been shown to have antioxidative and anti-inflammatory effects. Thus, polyphenols can reduce ROS production through oxidases inhibition, can reduce superoxide production, can inhibit OxLDL formation (48). Also, polyphenols through their antioxidant activity can modulate dyslipidemia offering protection against free radicals and thus decreasing the oxidation of the lipoproteins (Figure 3) (56).

**Figure 4:**
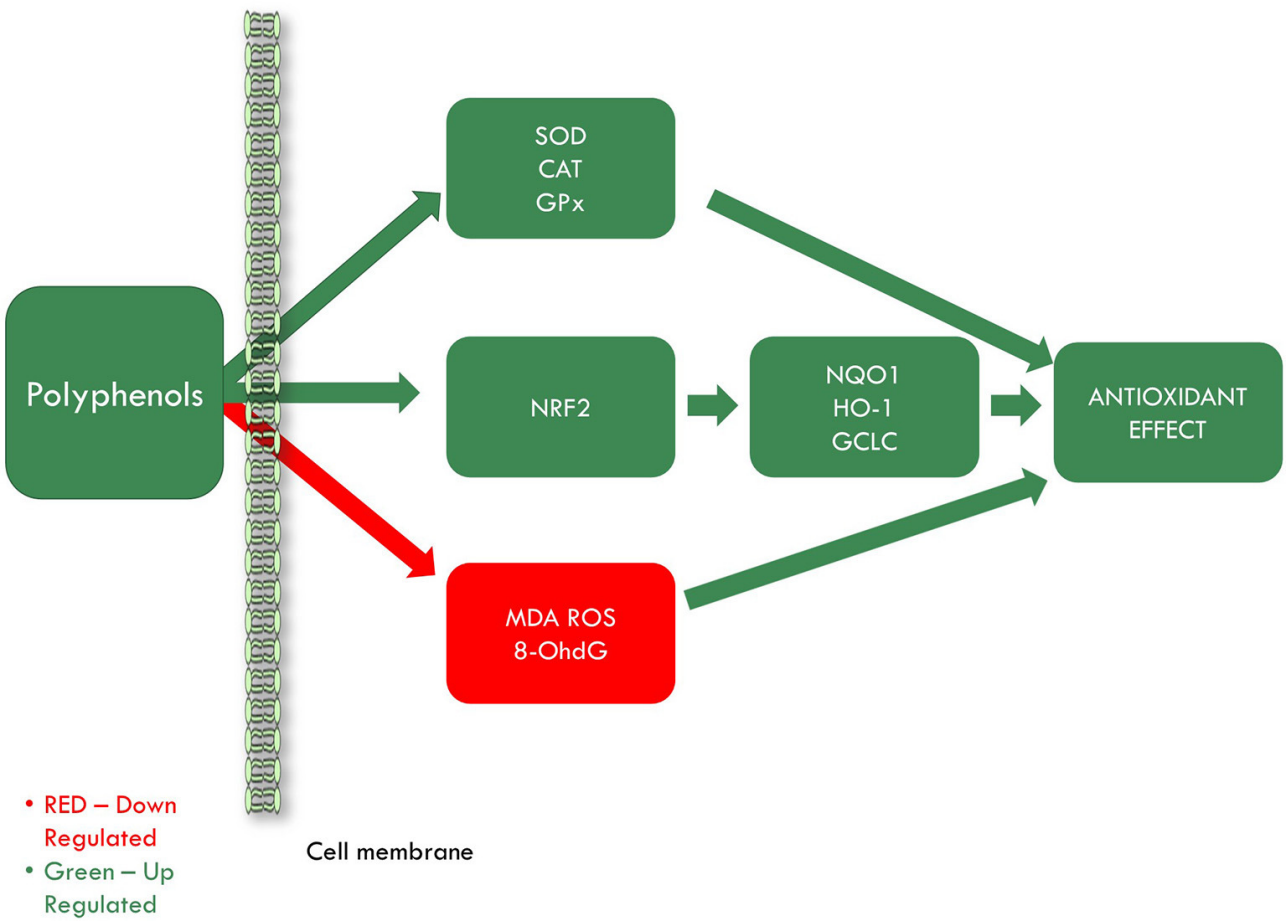
Grapevine and wine by-products antioxidant effect in ischemic heart disease. SOD, superoxide dismutase; CAT, catalase; GPx, glutathione peroxidase; NRF2, nuclear factor erythroid 2–related factor 2; NQO1, quinone oxidoreductase-1; HO-1, heme oxygenase 1; GCLC, glutamate-cysteine ligase catalytic subunit; MDA, malondialdehyde; ROS, reactive oxygen species; 8-OhdG, 8-hydroxy-2′-deoxyguanosine.

As seen, oxidative stress is a common process in many pathophysiological mechanisms underlying CVD. Polyphenols are known for their effect in reducing oxidative stress through their antioxidant activities (Figure 4). Much research is available regarding polyphenols antioxidant action. Even though most studies indicate that these compounds possess strong antioxidant activity, some studies are pointing out their pro-oxidant activity. That is why this review aims to discuss whether polyphenols contained in grapevine and wine by-products can be considered good candidates in the prevention of IHD in the light of their antioxidant action, pro-oxidant effect or their antioxidant action/pro-oxidant balance as demonstrated in the literature.

## Does Polyphenols From Grapevine and Wine By-Products Possess Antioxidant or Pro-oxidant Activity?

The antioxidant activity of phenolic compounds is described concerning their capacity to prevent the oxidation of carbohydrates, proteins, lipids, and DNA, generally at low concentrations (60, 61). Opposed, the pro-oxidant activity of polyphenols is described concerning their capacity to induce lipid peroxidation, DNA damage, mutagenesis, carcinogenesis, or apoptosis in cancer cells (61). It was already established that some phenolic compounds possess both antioxidant and pro-oxidant activity. These activities are in strong correlation with the administered dose, time, containing matrix as well as the presence of other components like metal ions. Cumulating these factors, the balance between beneficial and harmful effects can be very difficult to be controlled or predicted (27, 60, 62).

In general, polyphenols' action is described as being balanced in favor of keeping the organism's redox homeostasis (61). It was described that after ingestion, the polyphenols firstly act in the stomach then at the gastrointestinal tract level, and further in the blood system (61). Interestingly, it was observed that at the gastrointestinal level they act as reducing agents, while in the blood system, even at very low concentrations, they have pro-oxidant action through the generation of H_2_O_2_ or other oxidants capable to initiate oxidative stress processes (61). These processes may then be further identified in different organs, especially in the cardiovascular system, liver, pancreas, kidneys, lungs, or blood-brain barrier cells (61).

Therefore, it is very important to analyze if polyphenols act as both antioxidant or pro-oxidants and observe their health implications in more complex experimental settings that could potentially lead to the identification of both positive and/or negative effects in different organism structures.

Firstly, the positive health effects of GP or other polyphenols contained in wine grape by-products were tested on numerous *in vitro* studies that indicated both antioxidant or pro-oxidant action (Table 1). These studies raised multiple questions regarding their mechanism of action or the conditions affecting the balance from antioxidant toward pro-oxidant effect. Afterwards, several *in vivo* studies are verifying these hypotheses trying to establish a correlation between compounds concentrations and their effects and to identify their exact mechanism of action after being metabolized, in different organs (Table 1).

**Table 1:** *In vitro* and *in vivo* evidence of antioxidant and pro-oxidant effect of resveratrol and polyphenols from grapevine and wine by-products.

**Model**	**Sample**	**Antioxidant**	**Pro-oxidant**	**References**
***In vitro*** **models**				
- GRX cell line (hepatic stellate cell model): 24 h RSV treatment/120 h RSV treatment	- Resveratrol - Dose: 0.1, 1, 10, 50 μM	- Increased SOD activity (120 h treatment) - Reduced GSH levels (0.1–1 μM; 24 h treatment) - Reduced GSH levels (50 μM; 120 h treatment)	- Increased reactive species production (both treatments) - Decreased SOD activity (24 h treatment) - Decreased CAT activity (both treatments) - TBARS increased (both treatments)	(63)
- HT-29 - colorectal adenocarcinoma cell line - HCT116 - human colon carcinoma cell line	- Resveratrol – dose: 1, 10, 25, 50, 100 μM	- Increased cell number in HT-29 line (1, 10 μM RSV) - Increased SIRT1, SIRT6 expression (DNA repair)	- Decreased cell number in both lines (50, 100 μM RSV) - NADPH oxidase increased (50, 100 μM RSV) - Induced DNA damage (25, 50, 100 μM RSV)	(64)
- HT-29 - colorectal adenocarcinoma cell line	- Seasonings from GP seeds, GP skins, whole GP through different stage of digestion Dose: cell proliferation (100, 250, 500, 750, 1,000, 1,250, 2,500 μg) Dose: anti-genotoxic effect effect (200 μg/ml)	- Increased DNA protective effect The effect of GP prior digestion: GP skins> GP seeds and whole GPThe effect of GP after digestion:GP seeds and whole GP > GP skin	- Decreased cell proliferation in all fractions - GP skins (845 μg/ml) had higher effect than GP seeds (1,045 μg/ml)	(65)
- hGC- Human granulosa cells, KGN Human Granulosa-Like Tumor Cell Line	- Grape seed extract proanthocyanidin B2 Dose: 0, 0.01, 0.1, 1, 10, 50, and 100 μg/mL	- Decreased ROS (0.1, 1 μM) - Decreased NOX4 expression (0.1, 1 μM)	- Increased ROS (50, 100 μM) - Increased NOX4 expression (50, 100 μM) - Increased apotosis (50, 100 μg)	(66)
- FaDu, Detroit 562 cells – squamous cell carcinoma model	Grape seed extract Dose: 40 μg/ml	-	- Increased ROS formation due to impaired mitochondrial electron transport chain - Increased mitochondrial superoxide levels - Decreased GSH levels - Inhibited oxidative phosphorylation, glycolysis - Inhibited autophagy	(67)
- T24, HTB9 cells – bladder cancer model	- Grape seed extract (GSE) Dose: 25, 50, 100 μg/ml	- Increased autophagy	- Increased mitochondrial superoxide levels (GSE – 50 μg/ml) - Increased apoptosis in T24 cells (50, 100 μg/ml) - Increased apoptosis in HTB9 cells (25, 50, 100 μg/ml)	(68)
- Ca9-22 cells – gingival cancer model	- Grape seed extract - Dose: 1, 2, 4, 8, 10, 50, 100, 200, 400 μg/ml	-	- Decreased cell viability (50, 100, 200, 400 μg/ml) - Increased apoptosis (50, 100, 200, 400 μg/ml) - Increased DNA damage (50, 100, 200, 400 μg/ml) - Increased ROS levels (GSE – 50, 100, 200, 500 μg/ml)	(69)
Goat muscle cells subjected to H2O2 (pro-oxidant effect)	- Grape seed extract - Dose: 1, 10, 60 μg/ml	- CAT activity and gene expression increased (10 μg/ml) - SOD activity and gene expression increased (10 μg/ml) GPx activity and gene expression increased (10 μg/ml)	- No significant difference in TAOC at different concentrations of GSE - GSH reduced at 60 ug/ml GSE	(70)
PC-12 cells subjected to H_2_O_2_ (pro-oxidant effect)	- Grape Pomace Procyanidins - Dose: 25, 50, 100, and 150 μg/ml	- Cell viability increased (50, 100, 150 μg/ml) - MDA decreased (150 μg/ml) - SOD, GPx activities increased (150 μg/ml)	- MDA increased (50 μg/ml) - SOD, GPx activities decreased (50 μg/ml)	(71)
A549, H1299, H460, H322 cells – lung cancer model	- Grape seed extract - Dose used for A549, H1299: 50, 75, 100 μg/ml - Dose used for H460, H322: 20, 30, 50 μg/ml	-	- Increased apoptotic death in A549, H1299 cells (50, 75, 100 μg/ml) - Increased apoptotic death in H460, H322 (20, 30, 50 μg/ml) - Released cytochrome c, cleavaged capsases 3, 9 and PARP (apoptotic cascade) - Generated ROS in A549 (100 μg/ml), H460 (20 μg/ml) - Decreased GSH levels in A549 (100 μg/ml), H460 cell lines (20μg/ml) - JNK1/2 and ERK1/2 activated (oxidative stress markers) in A549 (100 μg/ml), H460 cell lines (20 μg/ml)	(72)
***In vivo*** **models**				
- 27 patients with stable COPD in a double-blind, randomized, placebo-controlled study	- Oligomeric proanthocyanidins - Dose: 150 mg/day	- Reduced the concentration of MDA - Decreased TC/ HDL-C ratio - Increased HDL-C	- Reduced the activity of SOD	(73)
- 80 ultraviolet radiation induced female SKH-1 mice	- *V. vinifera L*, Burgund Mare variety - Doses: 2.5 and 4 mg/cm^2^	- Increased SOD activity in groups treated with 2.5 and 4 mg/cm^2^ polyphenols after UV-B exposure; increased CAT levels in group treated with 4 mg/cm^2^ polyphenols - Reduced DNA damage in groups treated with 2.5 and 4 mg/cm^2^ prior UV-B exposure	-	(74)
- 24 high fat diet fed male Wistar rats	- Grape seed and skin from Tunisian *V. vinifera*- Dose: 4 g/kg	- Increased the activity of SOD - No statistically significant increased of catalase and GSH-Px activities	-	(75)
- 40 lead-induced hepatotoxicity male Wistar rats	- Grape seed procyanidin extract - Dose: 200 mg/kg	- Significantly reversed by reducing the MDA levels - Increased GSH levels - Increased SOD activity - Reduced ROS levels - Increased Nrf2 nuclear translocation directly and indirectly by suppressing miRNA153 and *via* AKT/GSK-3β/Fyn-mediated	-	(76)
- 223 *Nothobranchius guentheri* fishes	- Resveratrol - Dose: 200 μg/g food	- Reduced ROS levels - Up-regulated the activity of CAT - Increased SOD activity - Increased GSH-Px activity - Reduced oxidative damage: reduced protein oxidation, lipid peroxidation, decreased the accumulation of lipofuscin	-	(77)
- Caenorhabditis elegans strains wild type N2 (thermal stress at the 5th and 12th of adulthood)	- Four GP extracts from *V. vinifera L*. (Castilla y León, Spain): L1—fermented GP + hydroalcoholic mixtures (solvent), maltodextrin + silicon dioxide as encapsulation agents L2—fermented GP + hydroalcoholic mixtures (solvent), maltodextrin + silicon dioxide as encapsulation agents L3—GP + hydroalcoholic mixtures (solvent), maltodextrin as encapsulation agents L4—GP + water (solvent), maltodextrin as encapsulation agents	- 100 and 250 μg/mL L3 extract increased survival against oxidative damage - 250 μg/mL L1, L2 (only 12th day) and L4 extract increased survival against oxidative damage	- 1,000 μg/mL L3 extract decreased survival against oxidative damage	(78)
		- 1,000 μg/mL L2 extract decreased ROS levels on 12th day - 1,000 μg/mL L3 extract decreased ROS levels on 5th day - 100 and 250 μg/mL L4 extract decreased ROS levels on 12th day	- 1,000 μg/mL L2 extract increased ROS levels on 5th day - 100 and 250 μg/mL L3 extract increased ROS levels on 5th day - 1,000 μg/mL L3 extract increased ROS levels on 12th day	
- 24 1/2 Dorper × $\frac{1}{2}$ Small 78 thin-tailed crossed ram lambs (free vs. penned)	- Wine grape pomace (WGP) - Dose: 0, 5, and 10% WGP	- Reduced ROS and MDA levels at 5 and 10% WGP concentration - Increased mRNA expression of SOD and GPx4 at 10% WGP - No significant difference in mRNA expression of catalase and Nrf2 - 5 and 10% WGP increased the abundance of SOD and GPx4 protein - Only 5% WGP increased the abundance of catalase protein - No significant difference in the abundance of Nrf2 protein	-	(79)
- 36 Chios breed male sheep	- *V. vinifera L*. var. Moschato from Larissa, Greece - Dose: 9% GP	- No significant difference in SOD activity in liver and spleen - Significantly increased GST activity in liver and spleen - Significantly increased γ-GCS expression in liver - No significant difference in γ-GCS activity in spleen	-	(80)
- 24 piglets of Large White–Duroc–Pietrain × Landrace breed	- *V. vinifera L*. var. Moschato from Larissa, Greece - Dose: 9% GP	- Significantly decreased CAT activity at 50 days in erythrocytes - Significantly decreased CARB levels at 35 days in brain, spleen and liver and at 50 days in liver, quadriceps muscle, brain, spleen, lungs, stomach and pancreas - Significantly decreased TBARS levels at 35 days in heart, quadriceps muscle, brain, spleen, kidneys, lung and stomach and at 50 days in liver, heart, quadriceps muscle, spleen, lungs and pancreas - Increased TAOC levels at 35 days in pancreas and stomach and at 50 days in quadriceps muscle, kidneys, lungs, stomach and pancreas - Significantly increased GSH levels at 35 days in liver, heart, quadriceps muscle, brain, spleen, lungs, stomach and pancreas and at 50 days in liver, heart, brain, kidneys, lungs and pancreas - Increased H_2_O_2_ decomposition activity at 35 days in kidneys and at 50 days in quadriceps muscle, kidneys and pancreas	- Significantly decreased TAOC at 35 day in plasma and brain and at 50 days in brain - Decreased GSH levels at 50 days in spleen and stomach - Decreased H_2_O_2_ decomposition activity at 35 days in lungs and stomach and at 50 days in brain and lungs	(81)

*RSV, Resveratrol; SOD, superoxide dismutase; CAT, catalase; TBARS, Thiobarbituric acid reactive substances; SIRT, sirtuin; NADPH, nicotinamide adenine dinucleotide phosphate oxidase; GP, grape pomace; GSE, grape seed extract; ROS, reactive oxygen species; TAOC, total antioxidant activity; GSH, glutathione; GPx, glutathione peroxidase; HUVEC, human umbilical vein endothelial cells; MDA, Malondialdehyde; JNK, c-Jun N-terminal kinases; ERK, extracellular signal-regulated kinases; COPD, chronic obstructive pulmonary disease; OPCs, oligomeric proanthocyanidins; SOD, superoxide dismutase; MDA, malondialdehyde; GSH-Px, glutathione peroxidase; GSH, reduced glutathione; PF, polyphenols; ApoE-KO, apolipoprotein E knockout; ROS, reactive oxygen species; Nrf2, nuclear factor erythroid 2–related factor 2; AKT, protein kinase B; GSK-3β, Glycogen synthase kinase 3 beta; Fyn, a member of the Src-family protein-tyrosine kinases; CAT, catalase; GP, grape pomace; 8-oxodGuo, 8-oxo-7,8-dihydro-2′-deoxyguanosine (indicator of oxidative DNA damage); MDA, malondialdehyde; Nrf2, nuclear factor-like-2 factor; GPx4, glutathione peroxidase 4; GST, glutathione-s-transferase; γ-GCS, γ-synthase glutamyl cysteine (it is the first enzyme involved in the biosynthesis pathway of GSH); TAOC, Total antioxidant capacity; CARB, protein carbonyls; TBARS, thiobarbituric acid reactive substances; ALT, alanine trans- aminase; AST, aspartate transaminase; CK-MB, creatine kinase-MB*.

## *In vitro* Antioxidant/Pro-oxidant Activity of Polyphenols From Grapevine and Wine By-Products

The promising health benefits of GP, GS, or of its bioactive compounds including RESV contributed to the numerous *in vitro* studies conducted to investigate their potential as antioxidant agents using various models of cell lines.

The effect of grape seed proanthocyanidins extracts (GSPE), epicatechin (EC), gallic acid (GA), and RESV was investigated on a cytokine-induced vascular endothelial inflammation using a blood-brain barrier model. The endothelial damage, leptin receptors expression, and leptin transfer were investigated (82). Leptin is a hormone produced by adipokines that play a role in the metabolic system. Leptin is inhibiting appetite and hunger sensation in the central nervous system which in dietary obesity it cannot pass the blood-brain barrier due to existing low-grade inflammation. This fact can lead to fulnessless and weight gain (82). It was investigated whether phenolic compounds from grape could be beneficial for obesity by increasing the levels of leptin which can cross the blood-brain barrier. In this regard, a co-culture of rat brain endothelial cells, glial cells, and pericytes was used. Within the complex experimental settings, the effect of GSPE, EC, GA, and RESV against oxidative stress was also investigated., Their antioxidant capacity was measured after cellular incubation with IL-1β and TNF-α pro-inflammatory cytokines (82). By choosing the GSPE as a complex mixture of phytochemicals alongside EC as a flavonoid representant, RESV as a stilbene representant, and GA as a phenolic acid representant, an important overview on how the most important classes of phenolic compounds identified in grape by-products exert their antioxidant effects among others was obtained. Thus, GPSE and compounds' effect on ROS production, nitric oxide (NO) levels, NF-kB activation, and leptin transporter expression (Ob-Ra) were measured. It was observed that GSPE and RESV exhibited the highest antioxidant effect by reducing ROS levels, increasing NO levels, inhibiting NF-kB activation, and increasing Ob-Ra expression (82). Interestingly, on brain endothelial cells, GSPE increased NO production in both situations - when used alone and when used with the pro-inflammatory cytokines. Referring to EC and RESV, the NO levels were decreased when used in combination with the cytokines (82). This is an important observation related to phenolic compounds antioxidant /pro-oxidant activity especially to the one of GSPE. Within these experimental settings, the potential of GPSE to increase the NO production in both situations can lead to other hypotheses. Since NO is already known for its role as a regulator in numerous vital physiological functions (e.g., blood pressure, neural communication, and immune response) (83) we must ask if NO will act as an antioxidant or can have a pro-oxidant effect. Thus, the balance between a low concentration of NO known for the protective effect (antioxidant activity) and high NO concentrations (pro-oxidant activity) must be carefully controlled and modulated to induce the health-protective effects. These observations will need further research for new strategies in pharmacotherapeutic agents capable of maintaining antioxidant/pro-oxidant balance.

The effect of grape seed extract (GSE) and proanthocyanidin B2 on oxidative stress was also studied in an *in vitro* study using luteinized granulosa cells (hGC) and tumor granulosa cell line (KGN) (66). Regarding oxidative stress, ROS levels, and the NADPH oxidases 4 (NOX4) mRNA expression, a predominant ROS production enzyme found in hGC (66), were measured. It is known that GSE can have both positive or negative health effects in relation to NOX modulation (84). It was observed that low concentrations of GSE and proanthocyanidin B2 (0.1–1 μg/ml) reduced ROS levels but at higher concentrations (50 and 100 μg/ml) showed a significant increase of ROS. To support these results NOX4 mRNA expression was also tested and were found similar effects for both GSE and proanthocyanidin B2, with increased expression levels (66). Thus, this study demonstrates again the importance of antioxidant concentration in the induction of the antioxidant or pro-oxidant effect.

The capacity of GSE to modulate the antioxidant activity and its associated gene expression was investigated on a model of goat muscle cells subjected to the pro-oxidant effect of H_2_O_2_. Using different doses of GSE (0, 1, 10, 60 μg/ml—stressed group) or with GSE alone (unstressed group) it was investigated the antioxidant activity by measuring catalase (CAT), superoxide dismutase (SOD), glutathione peroxidase (GPx), glutathione (GSH) and total antioxidant capacity (TAC). CAT, copper-zinc superoxide dismutase (CuZn-SOD), and GPx-1 genes relative mRNA levels were also measured. Grape seed extract presented the capacity to regulate gene expression and activity of antioxidant enzymes. Total antioxidant activity was significantly increased in the unstressed group compared to one exposed to H_2_O_2_. In terms of GSH levels, this was not affected in the unstressed group. In the stressed group, H_2_O_2_ made the levels of GSH drop. A moderate dose of GSE (1, 10 μg/ml) not only didn't recover the previous levels of GSH but further increase in the dose (60 μg/ml) produced an even significant depletion in GSH levels. Enzyme activity and genes expression mRNA levels of SOD and CAT were also determined in both group cells. In the unstressed group, it was observed a significant drop-in activity of CAT and SOD compared with control. Corresponding changes in genes expression of these two enzymes (GSE-10 μg/ml) were also observed. In the stressed group, the activity of SOD and CAT rebounded to the level of the control group (10 μg/ml). mRNA levels of CAT showed a parallel growth with the activity of the enzyme and the same tendency was observed in the case of mRNA CuZn-SOD. GPx activity was also increased in the stressed group with levels increased back to the control levels (10 μg/ml). GPx-1 mRNA levels, had also rebounded to the level of the control group (70). Grape seed extracts show significant antioxidant activity in stressed cells by modulating the activity and expression of antioxidant enzymes.

Further, the antioxidant/ pro-oxidant balance of RESV, one of the main compounds in grapevine and wine by-products, was investigated on GRX cells exposed to different concentrations of RESV for different periods (24 and 120 h). It was observed an increase of ROS production in a time- and dose-dependent manner. The increase of ROS production caused a shortage in SOD activity, which was decreased after 24 h due to its consumption to convert ${\text{O}}_{2}^{-}$ into H_2_O_2_. After 120 h SOD activity was significantly increased in a time- and dose- pattern (63). Catalase activity was not significantly changed in the experimental groups (except for the group treated with 50 μM of RESV for 120 h). This observation may be significant for the outcome of the cells through regulation of the increased levels of H_2_O_2_. There were also changes in the dynamics of GSH levels (63). Treated cells with a low dose of RESV for 24 h and those treated with 50 μM of RESV for 120 h showed an increase in GSH levels (cells counterattack the pro-oxidant effect of resveratrol). Lipid peroxidation was also monitored through TBARS assay. After 24 h, 50 μM RESV showed a significant lipid peroxidation which lowered after 120 h (63). This change can be explained by the fact that RESV decreased the number of viable cells in the experimental groups and the remaining cells were the ones who adapted to oxidative stress conditions and continue division (*hormesis* concept) (63). The dual action of RESV on human colorectal adenocarcinoma (HT-29) and human colon carcinoma cells (HCT-116) was also observed. At a lower concentration of RESV (1 and 10 μmol/l), an increase in cell number occurred in HT-29 cell line but this effect diminished after the dose was increased to 25 and 50 μmol/l and even further at 100 μmol/l, resulting in a reduction in cell number (64). In the case of the HCT-116 cell line, there was not noticed a biphasic effect but a reduction in the number of cells based on the concentration of RESV. The effect of RESV on superoxide anions production and NADPH oxidase activity was also investigated. At 1 and 10 μmol/l, there was no significant modification in superoxide anions production or NADPH oxidase activity which correspond to the effect of RESV on cell proliferation observed in the first part of the study. At higher concentrations (50 μmol/l), NADPH oxidase activity was significantly higher which correspond to the concentration at which RESV exercised a decrease in cells number (64). To further investigate the pro-oxidant effect, the DNA damage and SIRT6 levels were assessed. By using histone γ2AX which is considered a DNA damage marker they showed that the level of DNA damage became significantly higher in a dose-dependent manner (25, 50, 100 μmol/l). Also by using SIRT6, enzymes with a role in antioxidant defense with a potential role in tumor growth inhibition in a low concentration, they showed that RESV had an opposite result, increasing the SIRT6 levels in a concentration-dependent manner (64). These observations were supported by some recent studies as well, done in different experimental settings. The role of RESV in oxidative stress damage was also investigated using TM3 mouse Leydig cells, cultured with H_2_O_2_. Here, RESV showed a protective effect for the Leydig cell (mitochondrial activity, metabolic activity, cell membrane, cell proliferation) at a low dose (10 μg/ml) but at a higher dosage revealed a tendency for negative effects. Superoxide production showed a significant decrease at low doses of RESV and a significant increase in production at high doses. Also, the steroidogenesis function was studied and the results showed biphasic results according to RESV concentration (85). The positive effect of RESV low dosage in oxidative stress damage was seen when applied to human trophoblast cells (HTR-8/SVneo) cultured with H_2_O_2_. The antioxidant enzymes like SOD and CAT showed a significant increase at a low concentration of RESV. The markers for cell membrane damage lactate dehydrogenase (LDH) and lipid oxidation (MDA) had a significant decrease when a low dose of RESV was given. Autophagy markers like Beclin-1 and LCR3II were also investigated to further analyze the antioxidant effect. It was observed that after cells incubation with RESV there was a significant decrease of their levels. The inflammation markers IL-1β and caspase-1 were also decreased in the presence of RESV (86).

## *In vivo* Antioxidant/Pro-oxidant Activity of Polyphenols From Grapevine and Wine By-Products

Since numerous *in vitro* studies already showed the potential health effects of these products but also underlined the importance between the concentration of these compounds and their influence in inducing both antioxidant and pro-oxidant effects, it is important to check whether this observation can be translated in *in vivo* studies as well. Thus, further, there will be discussed literature studies that can offer insights into the antioxidant/pro-oxidant processes within different experimental studies with a focus on how oxidative stress can affect different organs.

Accordingly, it was demonstrated that grape extract had beneficial effects against chronic seizures by its anti-oxidant action in the hippocampus, improving cognitive performance (87). Epilepsy represents, in a pathophysiological way, a pro-oxidant state in which oxidative stress, along with other mechanisms, plummets antioxidant substances, decreases antioxidant enzymes activity, increases ROS which affects mitochondria and that can trigger apoptosis cascade. Thus, the antioxidant effects of GSPE on rats with pentylenetetrazole (PZT) induced-epileptic seizures were studied. Ninety male rats were divided into 5 groups: control group (intraperitoneal saline injection for 60 days), PZT group (i.p saline injection 24 days followed by i.p 35 mg/kg/day of PZT for 36 days), PTZ + GSPE groups in which rats received GSPE (100 or 200 mg/kg/day) for 24 days before the first PZT administration and then pre-treated 30 min before everyday PZT injection for 36 days, and GSPE group who received 200 mg/kg/day for 60 days. After the treatment, they investigated the antioxidant effects of GSPE by measuring the levels of MDA, and GSH in the hippocampal homogenate and of hippocampus mitochondrial ROS. Cytochrome c, caspase 3, and 9 were also determined after 60 days, at the end of the treatment. In the epileptic seizure group treated with GSPE, there was a significant dose-dependent decrease in MDA levels and ROS compared to the PTZ group, but still at higher levels when compared to the control group. The level of GSH was also upregulated in a dose-dependent manner in the groups treated with GSPE when compared to the PZT group, but still reduced when compared to the control group (87). Thus, the results showed that GSPE can partly reduce the nocive effect of pentylenetetrazole. Since excessive ROS production can trigger apoptosis cascade, the levels of cytochrome c, caspase 3, and 9 were also determined. The levels of cytochrome c, caspase 3, and 9 were significantly lowered in epileptic seizure groups treated with GSPE as compared to the epileptic group and at higher levels when compared with the saline group indicating again the partly reverse antioxidant effect of GSPE (87). According to this *in vivo* study, GPSE is considered a safe intervention to attenuate the oxidative stress process in rat's hippocampus with chronic seizures. This study is also very important because it offers the perspective of the GPSE administration effect on some oxidative stress parameters like MDA, GSH, and ROS in normal physiological conditions. Since there were no significant differences between the saline group and GSPE group we can conclude that *in vivo* GPSE administration does not induce a pro-oxidant effect in rats hippocampus.

The antioxidant effect of GSPE on lead (Pb) -induced lung toxicity in rats was also investigated. Rats were divided into four groups: a control group who received saline, Pb (2,500 ppm of Pb acetate) group, GSPE (200 mg/kg) + Pb group, and GSPE group (88). To demonstrate the antioxidant effect of GSPE in Pb-induced lung toxicity, the levels of MDA, GSH, and SOD activities, and glutamylcysteine synthetase (γ-GCS) were determined. Also, for the study of oxidative stress, apoptotic rate, nuclear factor-erythroid-2-related factor 2 (Nrf 2), adenosine monophosphate-activated protein kinase (AMPK), heme oxygenase 1 (HO-1) expression, protein p62 levels, nuclear factor-κB (NF-κB), NAD(P)H quinone oxidoreductase 1 (NQO1) expression, tumor necrosis factor-α (TNF-α), Bcl-2 associated x protein (Bax), B-cell lymphoma-extra large (Bcl-xl), B-cell lymphoma-2 (Bcl-2) and p53 were determined (88). The group that received GSPE showed a significant decrease in MDA and ROS levels compared to the Pb group. The activities of SOD and γ-GCS also showed a significant increase in activity in the group of rats who received treatment with GSPE compared to the Pb group. For the study of apoptosis, lung apoptotic rate, pro-apoptotic and anti-apoptotic proteins were determined. In the GSPE + Pb group compared to the group who received only Pb, the apoptotic rate was significantly reduced, pro-apoptotic proteins significantly decreased (Bax and p53) and anti-apoptotic proteins significantly increased (Bcl-xl and Bcl-2). GSPE treatment significantly decreased NF-κB translocation in the nucleus and TNF-α expression. GSPE activity increased Nrf2 nuclear accumulation and increased NQO1, p62, and HO-1 levels compared with the Pb group. GSPE treatment showed increased activity of AMPK. The activation of AMPK can increase the accumulation of the Nrf2 in the nucleus which can lead to increase expression of NQO1, p62, and HO-1 and increased γ-GCS who can further increase the levels of GSH (88). Furthermore, HO-1 leads to decreased nucleus translocation of NF-κB and implicit, TNF-α expression. p62 is a protein with a role in protecting cells from oxidative stress. Increased levels of p62 by the Nrf 2 can lead to higher levels of Nrf 2 translocated to the nucleus through a positive feedback loop that further can increase the antioxidant response to the Pb-induced toxicity. Through the correlation of the results, it was concluded that GSPE reduced the toxic effects of Pb through AMPK/Nrf2/p62 pathway. Importantly, the fact that there were no significant differences between the group which received only GSPE and the control group, allows us to conclude that the antioxidant effects of GSPE manifest in the toxic environment without the induction of a pro-oxidant effect in rat lungs.

Moving down forward in the terms of reproduction, the protective effect of GSPE on apoptosis and oxidative stress was studied on a testicular dysfunction model induced by a high-fat diet (HFD) in rats. Forty male rats were divided into four groups: the control group (saline solution), HFD group, and HFD + GSPE groups (100 or 300 mg/kg) (89). The antioxidant activity of GSPE was determined by measuring GSH, GSH-PX, SOD, and MDA levels in the testicular homogenates. In terms of enzyme activity, the high-fat diet groups which received GSPE showed an increase in SOD and GSH-PX levels compared to the high-fat diet group. Also, the administration of GSPE in both concentrations increased the levels of GSH and decreased the levels of MDA when compared to the HFD group, suggesting GSPE's protective effect against the induced testicular oxidative damage (89). Moreover, administration of GSPE significantly decreased apoptosis in testicular germ cells when compared to the induced testicular toxicity group. However, when compared to the saline solution group, it was observed the tendency of GSPE to restore the levels of oxidative stress parameters to the normal conditions. Thus, at the highest dose of GSPE (300 mg/kg), there was no significant difference when compared to the saline group. These are suggesting that GSPE can attenuate the effect of induced oxidative stress through high-fat diet administration in testes tissues (89). Overall, the results of GPSE administration showed, besides an indirect effect by reducing body weights, several other direct effects: prevention in the drop of serum testosterone levels, reduction of testicular oxidative stress and germ cells apoptosis, and the attenuation of changes in spermatogenesis induced by a high-fat diet.

Furthermore, in a study realized on obese Zucker rats (model for genetic obesity), it was demonstrated that the treatment with GSPE has a beneficial effect on redox homeostasis by its antioxidant action on the liver (90). Thus, the effect of metabolic disorders on antioxidant reserves of the organism and the benefic effect of GSPE, specifically related to GSH hepatic metabolism was studied, considering GHS as a central defense response in oxidative stress prevention. Using 10 lean Zucker rats (control group treated with diluted sweetened condensed milk as a vehicle) and 20 fat Zucker rats divided into two groups: obese Zucker rats (OZ) feed with the vehicle and obese Zucker rats treated with GSPE (vehicle and 35 mg GSPE/kg body weight/day) (90). To assess the antioxidant capacity of GSPE, the GSH metabolism (glutathione/ oxidized glutathione (GSH/GSSG) ratio), the antioxidant enzymes activities (GPX, GR, and GST activities), lipid peroxidation (MDA), and hepatic ROS levels were determined in the liver tissue. In addition, TAC was determined in rat's plasma. The results of these analyses were discussed also in correlation with the mRNA expression of the investigated antioxidant enzymes like glutamate-cysteine ligase catalytic subunit (Gclc), GR, GPx, and GST as well as of Cu, Zn-SOD. It is known that Gclc is responsible for GSH synthesis, GR for the reduction of GSSG to GSH in an NADPH-dependent manner, GPx for lipid protection against peroxidation, GST for cellular detoxification, and Cu,Zn-SOD acts as a scavenger of superoxide anions sustaining the oxidant/antioxidant steady-state (90). The results indicated that GSH/GSSG ratio had increased levels in OZ + GSPE compared to the OZ group, suggesting the protective effect against oxidative stress production in the liver. In the case of the enzymes that regulate the levels of GSH and GSSG, some modifications deserve mention. Accordingly, it was observed that in the OZ + GSPE group Gclc mRNA expression and GSH levels had no significant difference when compared to the OZ group. Concerning GR mRNA expression, it was observed that even if GR expression was not decreased, the enzyme activity was decreased in the OZ + GSPE group (lower than the control group). The GPx relative expression showed a significant reduction in the OZ + GSPE group compared to the OZ group while GPx enzyme activity had significantly higher levels in OZ and OZ + GSPE groups when compared to control (90). Finally, the GST mRNA expression suffered no modifications among groups while GST enzyme activity had significantly lower levels in the OZ + GSPE group as compared to the OZ group. Further, regarding MDA and ROS levels, there was a significant decrease of MDA in OZ and OZ + GSPE groups and no significant changes between any group in the case of ROS. Also, Cu,Zn-SOD mRNA expression had no significant modifications in the OZ or OZ + GSPE group. This study is very important since it correlated the mRNA expression of some important antioxidant enzymes involved in liver GHS alteration with their corresponding enzyme activities and it allowed to provide insights regarding the GSPE mechanism of action. Thus, it can be observed that GSPE treatment can directly lower some of the enzymes activities (GST, GPx, and GR) by their direct antioxidant effect and not through the activation of the cellular antioxidant defense (90).

The beneficial effects of GSPE were also tested on mouse liver, using an iron overload-induced fluoride model (91). In this regard, forty rats were divided in four groups: control group (distilled water), natrium fluoride (NaF−100 mg/l) group, NaF + GSPE (400 mg/kg body weight) and GSPE group. For oxidative stress assay, the levels of glutathione peroxidase (GSH-Px), TAC, SOD, and MDA were measured (91). To determine iron overload, the iron levels in the liver were determined as well as the levels of mRNA expression and protein levels of hepcidin and ferroportin. It is known that iron in excess can be cytotoxic and can produce oxidative stress through the Fenton reaction in which hydroxyl radicals are formed. Hepcidin is the key role protein in the regulation of iron metabolism. The hepcidin production is regulated by iron stores and when the iron stores are higher than physiological value more hepcidin is produced and iron is sequestered in the cells through internalization and even destruction of ferroportin, a protein with a role in externalizing iron from the cell. Once is being captive in the cells, excess iron can produce oxidative stress through the Fenton reaction (91). Thus, the iron levels significantly dropped in the group treated with GSPE when compared to the NaF group (91). The hepcidin mRNA and protein expression were significantly lower in the group treated with GSPE compared to the NaF group. Ferroportin mRNA and protein expression were significantly higher in the group treated with GSPE compared to the NaF group. In terms of oxidative stress, the treatment group (NaF + GSPE) showed significantly increased levels of GSH-Px, TAC, and SOD compared with the NaF group. The MDA levels were significantly lower in the NaF + GSPE group compared with NaF. Even if the GSPE showed significant antioxidant activity compared with the NaF group, the levels were still not comparable with the control group. This study also supports the previous research' findings regarding GSPE treatment in healthy rats. Since there was not a significant difference between the GSPE group and the control group we can conclude that GSPE did not induce pro-oxidant effects and its antioxidant effects were manifested only in oxidative stress conditions.

So far the results of *in vivo* studies suggest that GSPE administration does not induce a pro-oxidant effect on rats hippocampus, lungs, and liver when administrated in normal physiological conditions and its antioxidant effect occurs only when the oxidative stress was experimentally induced.

## *In vitro* and *In vivo—*Antioxidant/ Pro-oxidant Balance of Polyphenols From Grapevine and Wine By-Products

In this context, before the *in vivo* experiments, the presence through absorption of polyphenols derived from GP as well as the antioxidant/pro-oxidant balance generated by those was investigated in an *in vitro* study on IPEC-1 cells and further an *in vivo* study in the piglets' duodenum and colon (30). To establish the best correlation between *in vivo* and *in vitro* settings, the antioxidant or pro-oxidant activities of phenolic compounds were indirectly investigated by UV-VIS spectroscopy. It was demonstrated that oxidation occurs when a bathochromic shift of λmax was observed in spectral assessment. Both extracellular and intracellular matrices were analyzed. During the *in vitro* experiment, it was observed that the highest dose of GP extract produced a λmax bathochromic shift already after 3 h of treatment, and after 24 h in case of the lowest dose, suggesting that the oxidation of the GP extract is time and dose-dependent (30). Interestingly, in the intracellular matrix, the GPE treatment did not affect it after 3 h, but after 24 h a bathochromic shift was observed only at the highest dose, indicating slight oxidation of polyphenolic molecules. Further, within the *in vivo* studies, the λmax of the polyphenols identified in the colon and duodenum was shifted, indicating slight oxidation after ingestion (30). The antioxidant activity of colon and duodenum tissues was also investigated following lipid peroxidation (TBARS), Total Antioxidant Status (TAS), CAT, SOD, and GPx. It was observed that piglets diet supplementation with 5% GP had a differential effect according to the investigated organs. Thus, TAS was increased and TBARS was decreased in both duodenum and colon, while SOD activity was increased only in the duodenum and CAT and GPx activity only in the colon (30).

Further, GP antioxidant and pro-oxidant activity were evaluated both *in vitro* and *in vivo* using a different approach. If the previous study used UV-Vis spectroscopy as the main method of evaluation of antioxidant/pro-oxidant activity, within this study, cyclic voltammetry assay was used to establish the *in vitro* antioxidant or pro-oxidant potential of polyphenols compounds found in the GP extract. This method evaluates both antioxidant and pro-oxidant activity of the extracts at the same time, indicating, for a mixture of different extracted phenolics the potential to act as antioxidant or as pro-oxidant (14). Thus, extracts are characterized simultaneously in terms of anodic oxidation potentials. Accordingly, low oxidation potentials are describing the antioxidant activity of the extract, whereas the high oxidation potentials are describing the pro-oxidant activity (14). It was observed that *in vitro* antioxidant and pro-oxidant activity are depending on the extraction method and grape varieties. Also, the presence in different mixtures of different phenolic classes are influencing the radical scavenging activity through the OH substitution or conjugation patterns on the skeleton of the compound (14). A different approach was then further applied during the *in vivo* study. Using the phytopathogenic fungus *Botrytis cinerea*, different GP mixtures previously assayed by the cyclic voltammetry method were chosen to see their influence on mycelial growth. By measuring the accumulation of ROS it was established the pro-oxidant effect of the extracts on B. *cinerea* mycelial growth. Overall the *in vitro* antioxidant activity with *in vivo* assays could not be correlated (14).

The effect of grape seed extract on the modulation of antioxidant and pro-oxidant balance was investigated on different yeast strains with the compromised antioxidant system (e.g., deleted cells in SOD, CAT, GSH synthase and simultaneously knockout in old yellow enzyme 2 and glutathione reductase 1). This was performed to simulate the most common cellular dysfunctions encountered in the oxidative stress process. Thus, programmed cell death was induced through a known concentration of H_2_O_2_ in four deleted cells. When compared the untreated with treated wild-type yeast strain (BY4741), treatment with GSE (50 uM gallic acid equivalents of GSE) had a significant effect (12% enhancement) on cellular growth. Interestingly, at a lower concentration (5 uM gallic acid equivalents of GSE) the extract had a pro-oxidant effect (92). Regarding the deleted cells the GSE positively influenced the growing proliferation in CAT or GSH deleted cells indicating the extract potential involvement in the intracellular peroxide concentration, membranes oxidative damage, or GSH metabolic pathway (92). The pro-oxidant effect was observed in cells deleted in SOD activity, the production of ROS levels varying in a dose-dependent manner (92). Thus, the results gave an important perspective on the *in vivo* manifestation of antioxidant/pro-oxidant effect which according to their experimental setting is depending on the cellular antioxidant system deficiencies.

The pro-oxidant activity of polyphenols from red wine was studied on human erythrocytes in an *ex vivo* study (93). It was demonstrated that polyphenols from red wine can activate the plasma membrane redox system that is involved in the neutralization of plasma free radicals by changing the GSH pathway *via* intracellular ROS production. The erythrocytes from 10 healthy donors were incubated with red wine polyphenols in a concentration similar to the one obtained after regular consumption of red wine for 2 weeks. It was observed that red wine polyphenols increased ROS concentration which generated a pro-oxidant effect. Importantly, by measuring the aldose and NADH-methaemoglobin reductase activities, it was observed that the induced pro-oxidative process had no harmful effect on the erythrocytes. The aldose reductase is an enzyme responsible for multiple physiological roles including aldehydes detoxification (93). The NADH-methaemoglobin reductase offers insights regarding the toxicokinetic of methemoglobin reduction, indicating the oxidation of hemoglobin from ferrous (Fe2+) to the ferric (Fe3+) state, a form that is unable to bind oxygen (94). Thus, it was concluded that the anti-oxidant effect was not connected to any harmful effect, since no significant differences were observed between treated and untreated cells (95). This induced the adaptive stress-response translated in erythrocytes protection against numerous adverse conditions considering that moderate induction of the oxidative stress can stimulate cell defense systems activation (95). This is an important study that highlight that even if polyphenols compound in grape wine act as pro-oxidants agents, they, in turn, trigger the erythrocyte adaptive cellular response with a final protective effect.

It was also demonstrated that on primary leucocyte culture a GSE had an antioxidant activity mediated by the polyphenols pro-oxidant oxidation products and *o*-quinones. Even though the flavan-3-ols are well-known for their antioxidant activity, they can also become pro-oxidant because they can be oxidized to *o*-quinones or semiquinones, which further initiate the redox cycling and reactive oxygen species generation, and thiol, DNA, and protein alkylation (62, 96). While the simple catechin *o*-quinone are pro-oxidants, the procyanidin *o*-quinones form oligomeric compounds with the same number of hydroxyl groups through the same futile redox cycling process (97). It was also indicated that the procyanidins are superior antioxidants compared to monomeric catechin which is more easily prone to enter the redox cycle and generate a pro-oxidant action (97). *O*-quinones, on the other hand, are expected to have an antioxidant action because they can be reduced back to diphenols through semiquinones (98). Catechin *o*-quinones can react *via* phenolic coupling forming dimers and oligomers. Each formed molecule has its original reactive hydroxyl groups, thereby increasing their antioxidant properties until a certain level when the oligomers will precipitate becoming insoluble (98). This can explain how the delicate antioxidant/pro-oxidant balance is tuned through *o*-quinones. It was also demonstrated that *o*-quinones are involved in the pro-oxidant behavior of quercetin and caffeic acid mixture (99).

## Ischemic Heart Diseases: Current Treatment and Limitations

Coronary artery disease can be characterized by long, stable periods or at some point can become unstable. Generally, the instability can be caused by an acute atherothrombotic event as a result of plaque rupture or erosion. However, CAD is considered a chronic and progressive disease, that according to its dynamic can be conveniently classified as acute coronary syndromes (ACS) or chronic coronary syndromes (CCS) (100). The clinical manifestation of CAD is angina pectoris, typically observed in the chest, neck, or left arm, arrhythmia, myocardial infarction and heart failure (58). The clinical manifestation of chronic angina can double the risk for important cardiovascular events (101). Treatment of patients with IHD has as primary goal to maximize the quality of life and further reduce the mortality risk. According to the imposed clinical situation, the management of IHD is realized *via* medical, interventional, and even surgical treatment. The initial treatment for patients with angina pectoris and persistent elevated ST segment (STEMI) is focused on patients' pain, dyspnea, and anxiety. Thus, potent antalgic drugs like opioids (morphine), oxygen through masks for dyspnea and benzodiazepines are used to reduce anxiety. The gold standard for STEMI patients is the percutaneous coronary intervention (PCI) within 2 h from the diagnose of the myocardial infarction and pharmacotherapy like antiplatelet (aspirin and either prasugrel or ticagrelor) and anticoagulant (unfractionated heparin, enoxaparin) drugs. If PCI cannot be performed in time, fibrinolysis treatment should be considered: alteplase (t-PA), reteplase (r-PA), or tenecteplase (TNK-tPA) in combination with antiplatelet and anticoagulation drugs (102).

On the other hand, long-term pharmacotherapy includes the utilization of several drugs that are aiming to reduce, control or stop the progression of CAD. Thus, the decrease in oxygen demand and the increase in oxygen supply are achieved by nitroglycerines that induce coronary artery dilatation (58). The stabilization of atherosclerotic vascular plaque is achieved by the use of statins, renin-angiotensin-aldosterone system (RAAS) inhibitors, and antiplatelets (acetylsalicylic acid, clopidogrel) through their pleiotropic antioxidative effects (103). Statins are also important in reducing cholesterol, improving endothelial function, blood vessels dilatation, and have anti-inflammatory effects. Aspirin and clopidogrel (as anti-platelet agents) are used to lower the atheromatous plaque formation by inhibiting platelet aggregation. Further, angiotensin-converting enzyme (ACE) inhibitors, are used to decrease myocardial oxygen consumption, increase myocardial blood flow and decrease the risk for recurrent myocardial infarction. Calcium channel blockers are used to lower the intracellular calcium level, to relax vascular smooth muscle, and to dilate the blood vessels (58). Beta-blockers can also be used to reduce myocardial oxygen consumption.

No matter the approach, the incidence of deaths and myocardial infarction due to CAD complications is still far from being at an acceptable rate.

Overall CAD management can relieve symptoms and reduce the risk of complications, but long pharmacotherapy term use of CAD can also come with multiple side effects. Antiplatelets can increase the risk of bleeding (clopidogrel) or induce stomach ulcers (acetylsalicylic acid). Statins can induce hepatic toxicity *via* moderate elevation of aspartate aminotransferase (AST) and alanine aminotransferase (ALT) serum levels, can get muscle pain or rhabdomyolysis (104). Beta-blockers can decrease blood pressure or heart rate too much. ACE inhibitors can also lower blood pressure too much and may also induce dry cough (104). These are the most common side effects that most of the time are solved by dose adjustments.

But irrespective of how revascularization techniques and drugs effectiveness is, a healthy lifestyle and appropriate diet management should not be ignored since these factors can initiate and also worsen CAD prognostic. Thus, for long-term treatment the patient should be more cautious to reduce the risk factors (e.g., quit smoking, reduce blood pressure, control body weight, have a healthy diet, physical activity) (105).

That is why new pharmacological alternatives may be able to both reduce side effects and decrease risk factors for IHD treatment are of great interest. This is the loophole where polyphenols, taken as adjunctive therapies can have a positive impact in reducing CAD risk factors through their synergistic effects.

## *In vitro* and *In vivo* Studies—Antioxidant/Pro-oxidant Balance of Polyphenols From Grapevine and Wine By-Products: Focus on IHD

**Figure 5:**
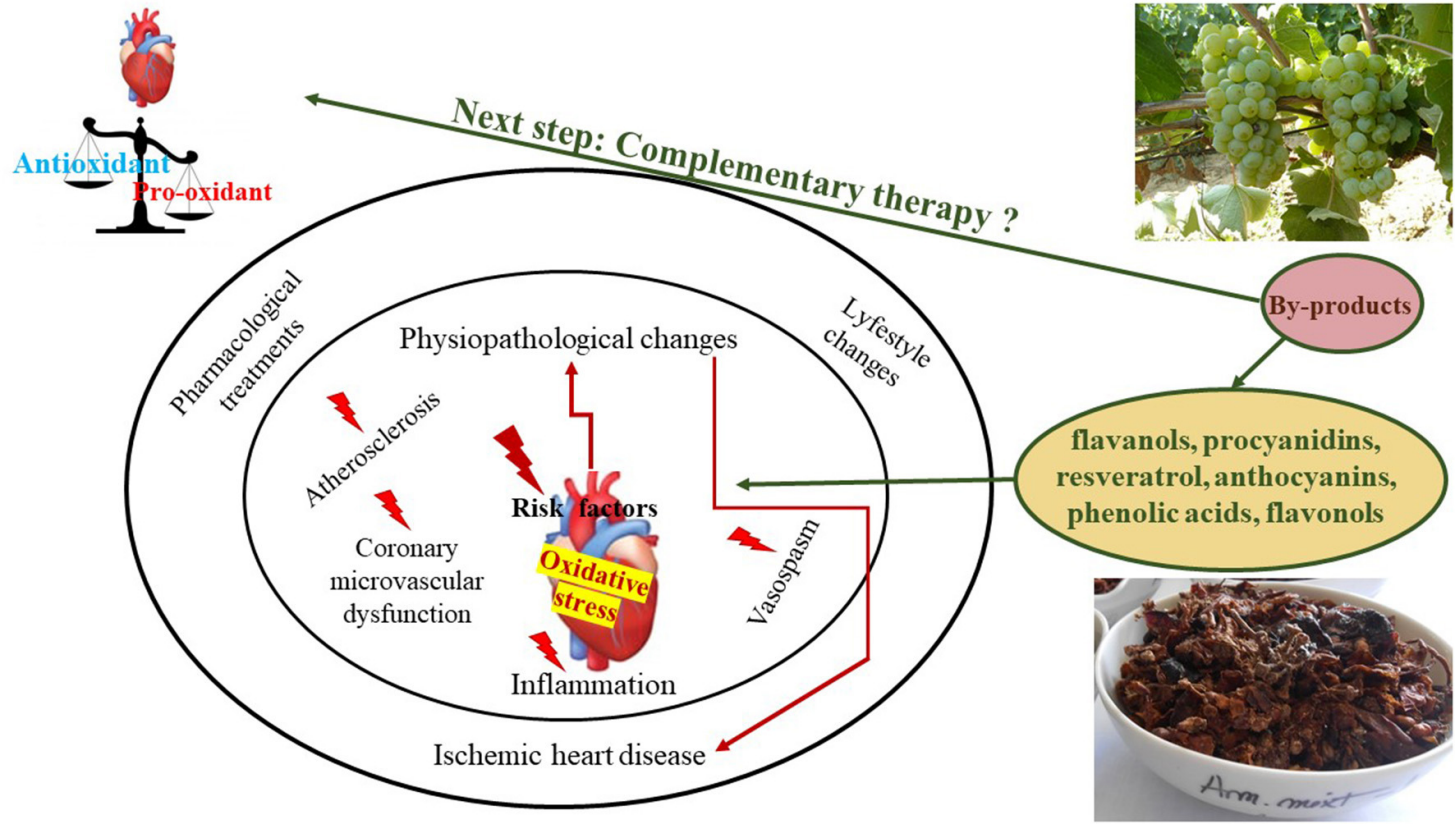
Grapevine and wine by-products antioxidant/pro-oxidant influence on ischemic heart disease risk factors.

It is known that organisms are exposed to ROS, or reactive oxygen metabolites like superoxide anions (•${\text{O}}_{2}^{-}$) hydrogen peroxide (H_2_O_2_), or hydroxyl radicals (•OH) as resulted of the oxidative metabolism or by exposure to the radical-generating compounds (106, 107). Also, free radicals and ROS were studied for their role in the different development stages of various diseases and how these are affecting the target organs. So far, as previously discussed, GP and grapevine by-products have proved to protect against multiple stress injuries by their antioxidant effects in various *in vitro* and *in vivo* models (Table 2). Moreover, their consumption proved to be safe and showed a protective effect against induced oxidative stress in various organs like testes, liver, kidneys, colon, duodenum, hippocamp. Their consumption has been shown to manifest a protective effect on the cardiovascular system mainly through their hypolipidemic, hypotensive, and anti-atherosclerotic effects (23, 121, 122). Also, it was demonstrated their effectiveness in improving plasma antioxidant status (122). Through different experimental studies, it was shown that polyphenols could reduce atherosclerosis by inhibition of LDL oxidation or other cellular redox states, by improvement of endothelial function, by lowering blood pressure, by inhibition of platelet aggregation, by reducing inflammation, or other mechanisms (Figures 3, 5) (123). Special attention should be directed toward the anti-atherosclerotic effects since it was already demonstrated that while atherosclerosis is a key factor involved in the process of CAD development and progression, the oxidative modifications within the arterial wall are directly involved in the initiation and/or contribution of the atherogenesis (Figure 3) (124).

**Table 2:** *In vitro* and *in vivo* evidence of antioxidant effect of resveratrol and polyphenols from grapevine and wine by-products.

**Model**	**Sample /dose**	**Antioxidant effects**	**References**
***In vitro*** **studies**			
- Human neonatal cardiomyocyte (HCM) treated with endotoxin lipopolysaccharide (LPS)	- Resveratrol - Dose: 3 μM for 4 h	- Significantly reduced mitochondrial ROS production	(108)
- Neonatal rat cardiac cells treated with fractalkine with/-out pre-treatment with Resveratrol subjected to H_2_O_2_ - induced oxidative stess or anoxia/reoxygenation cycle	- Resveratrol - Dose: 25 μM	- Down-regulated ICAM-1, MMP-9, ANP, TGF-β, FKN, procollagens I and III - Preserved cardiac cells viability - Enhanced cardiac cells autophagy	(109)
- Neonatal cardiac cells exposed to ischemia/reperfusion	- Resveratrol - Dose: 100 μmol/L	- Increased cell viability; Bcl-2 expression, SOD levels - Decreased LDH activity, apoptotic rate, capsase-3 activity, Bax expression, MDA levels - Increased Na+-K+ -ATPase and Ca2+ -ATPase activity	(110)
- Human cardiomyocytes (HCM) azidothymidine-induced cardiotoxicity	- Resveratrol - Dose: 3 μM	- Reduced cardiomyocytes apoptosis - Reduced the activity of caspase-3 and caspase-7 - Reduced mitochondrial ROS generation	(111)
- H_2_O_2_ exposed cardiomyocytes extracted from Sprague Dawley rats	- Pre-treatment with Resveratrol (doza) - Dose: 1 mL at 2.5 mg/kg oral gavage	- Prevented this reduction of SOD and CAT activity	(112)
***In vivo*** **studies**			
−20 male albino Wistar rats with isoprenaline induced myocardial infarction	- *V. vinifera* L. var. Fetească neagră fresh GP extract (FNFs) - Dose: 1 mL/day - Fermented GP extract (FNFr) from Mure? county, Romania - Dose: 1 mL/day	- EKG – protective effects - FNFs reduced RR interval, QT interval, ST depression and increased the heart rate - FNFr reduced RR interval, ST depression and increased the heart rate - FNFs and FNFr significantly reduced ALT, AST, and CK-MB levels	(113)
−60 mices with induced ischemic heart disease (atherogenic diet)	- Red wine grape pomace - Dose: 10% grape pomace	- Significantly reduced IL-10, TNF-α levels - Significantly increased HDL cholesterol - Decreased dimension and number of atherosclerotic lesion - Restored ejection fraction in left ventricle	(114)
- C57BL/6 male mice endotoxin-induced cardiomyopathy	- Pre-treatment with Resveratrol - Dose: 10 mg/kg intraperitoneal injection	- Significantly reduced the elevation of CK and LDH	(108)
- C57BL/6 mice induced myocardial infarction through cardiac surgery	- Resveratrol - Dose: 20 mg/ kg/day intraperitoneal injection for 42 days	- Increased survival rate - Increased left ventricular function - Decreased left ventricular infarct size	(109)
- Langandorff Model – *ex vivo* technique, treated with fractalkine and with/-out Resveratrol	- Resveratrol - Dose: 25 μmol/l	- Decreased infarct size	
−32 male Rattus Norvegicus rats with induced ischemia by placing tissue from the left ventricle in transport solution (0.09% NaCl) at 4°C for 5 h	- Grape seed proanthocyanidin - Dose: 100 mg/kg twice a day	- Decreased ischemia-related MDA - Increased levels of SOD, CAT and GPx activity	(115)
- Streptozotocin induced diabetes and isoproterenol induced infarction in male Wistar rats	- *V. vinifera* seed methanolic extract - Dose: 125 or 250 mg/kg	- Significantly reduced levels of LPO - Significantly reduced RAGE protein expression - Reduced levels of TNF-α, NF- κβ, Iκκβ, IL-1β, and IL-6 - Significantly increased levels of SOD, CAT and GPx - Increased activity of Na(+)/K(+)-ATPase and Ca(2+)-ATPase	(116)
−24 male Wistar rats arsenic-induced cardiac oxidative stress and injury	- Grape seed and skin extract (GSSE) of V. *vinifera* (doza) - Dose: 4 g/kg	- Increased activity of CAT, SOD, and GPx - Decreased free iron, H_2_O_2_ and ionizable calcium levels	(117)
L-NAME-induced malignant hypertension rats	- Resveratrol - Dose: 10 mg/kg/day	- Decreased TBARs, superoxide anion radical, thiol groups, MPO - Increased NO_2_ - Prevented reduction of antioxidant enzymes (CAT, SOD, GRx, GPx) - Reduced cardiomyocyte damage score - Reduced TGF-β expression	(118)
Male rats ischemic/reperfusion injury	- Resveratrol - Dose: 2.5 mg/kg/day for 15 days	- Reduced ROS - Increased GSH - Reduced infarct size - Increased aortic flow, left ventricular development pressure post-ischemia	(119)
- Heart from male ApoE-KO mice	- Resveratrol - Dose: 30 or 100 mg/kg for 7 days	- No effect on NOX1 genes - Significantly reduced the expression of NOX2 and NOX4 - Reduced ROS/superoxide levels - Increased the expression of SOD (isoform SOD1, SOD2, and SOD3), GSH-Px, and catalase	(120)

*ROS, reactive oxygen species; ICAM-1, intercellular adhesion molecule 1; MMP-9, Matrix metallopeptidase 9; ANP, Atrial natriuretic peptide; TGF-β, transforming growth factor beta; FKN, fractalkine; SOD, superoxide dismutase; LDH, Lactate dehydrogenase; MDA, malondialdehyde; CAT, catalase; ALT, alanine transaminase; AST, aspartate transaminase; CK-MB, creatine kinase-MB; IL-10, interleukin-10; TNF-α, Tumor necrosis factor α; HDL, high density lipoprotein; GPx/GSH-Px, glutathione peroxidase; LPO, lipid peroxidation; NF-κβ, nuclear factor kappa-light-chain-enhancer of activated B cells; Iκκβ, inhibitor of nuclear factor kappa-B kinase subunit beta; IL-1β, interleukin-1β; IL-6, interleukin-6; TBARs, thiobarbituric acid reactive substances; MPO, myeloperoxidase; GRx, glutathione reductase; GSH, glutathione*.

The effect of red wine GP on atherosclerosis and myocardial damage was studied using a lethal model of IHD on SR-B1 KO/ApoER61h/h mice, fed with an atherogenic diet (114). The mice were divided into three groups. The first group was fed with the high cholesterol atherogenic diet with 20% of total diet of control chow diet (control group), the second with 20% RWGP flour (GP group), and the third with 10% chow/10% oat fiber (the positive control group) (114). Regarding the oxidative stress status, plasma was used to investigate HDL-containing plasma antioxidant activity and MDA levels, while aortic roots for the investigation of the atherosclerosis process. The levels of inflammatory markers like tumor necrosis factor α (TNF-α) and interleukin 10 (IL-10), total lipoprotein, and total cholesterol were also assessed. It was observed that food supplementation of the atherogenic diet with red wine GP increased the antioxidant activity of plasma containing HDL particles after 14 days of treatment, suggesting its effect on lipid peroxidation modulation (114). Also, red wine GP supplementation significantly decreased the induced atherosclerotic lesions as compared to the control group as seen after the aortic root cross-sectional area analysis. The red wine GP supplementation had a positive impact on myocardial dysfunction and reduced the myocardial infarction after 14 days of supplementation, the risk of macroscopic IHD being reduced by 86% (114). This study strongly suggests that red wine GP supplemented *via* food intake can have a positive effect on atherosclerosis development associated with an increased plasma antioxidant capacity (114). Thus, it was considered a strong adjuvant in the prevention of ischemic cardiovascular events, in disease development and progression.

Further, the antioxidant properties, as well as endothelium-dependent vasodilatation effect of an enzymatic GP extract, were tested for the vascular reactivity using intraperitoneally and thoracic aorta and small mesenteric arteries isolated from male Wistar rats (125). The NO involvement in GP effect on the vascular response has been assessed. Grape pomace effect on the aorta and small mesenteric arteries contraction was determined after SOD or endothelin-1 exposures, known for its capacity to induce ${\text{O}}_{2}^{-}$ through NADPH oxidase activation (125). Afterwards, the arterial ${\text{O}}_{2}^{-}$ production was determined to evaluate ROS's role on the vascular effects. The concentration of phenolic compounds in GP used to treat rat arteries ranged from 0.0001 to 0.03 g/L. It was observed that the extracts had a relaxation effect in the aortic rings in a dose-dependent manner. The relaxation effect was attributed to endothelial nitric oxide synthase (eNOS) activation following a NO-dependent mechanism (125). Grape pomace extract reduced ${\text{O}}_{2}^{-}$ production at only 0.03 g/L. This observation is important since it gives important information related to the concentration of phenolic compounds needed to inhibit NADPH oxidase, the main source of ${\text{O}}_{2}^{-}$ production in the vascular tissues. Accordingly, the inhibition effect was obtained at three-fold lower concentrations than previously determined scavenging properties obtaining a reduction of 50% in the DPPH assay (125).

The antioxidant and cardioprotective effect of GPE was studied on isoproterenol-induced myocardial ischemia in rats (113). Twenty rats were divided into four groups: the control group, which received a saline solution, the positive control group that received a saline solution, and isoproterenol (150 mg/kg, subcutaneous injection) on the 8th and 9th day. The other two groups received instead of saline, GPE of fresh or fermented GP, and isoproterenol following the same conditions (113). Fermented GP was used since it is known that the fermentation process releases an increased amount of polyphenols from GP. The cardioprotective and antioxidant effects of GPE were analyzed considering the electrocardiography results, the oxidative stress and cardiac markers serum results. The results of the electrocardiography indicated that fresh GPE had better cardioprotective results as expressed by reduced RR, QT, and increased QTc, HR increasing, and reduction of ST depression as compared to the positive control group (113). Further, knowing that in myocardial infarction, the myocardial injury is mainly caused by the imbalance in the oxidant and antioxidant defense system (113, 126), the oxidative stress was investigated following the next parameters: TOS, total antioxidant response—(TAR), oxidative stress index—(OSI) as the ratio between TOS and TAR, MDA, total thiols (SH) and NO synthesis (NOx). The GPE pre-treatment significantly reduced TOS and OSI levels while increasing TAR, opposite to results from the positive control groups. Also, MDA production was significantly decreased in GPE groups as compared to the isoproterenol group. No effect was observed on SH and NOx production (113). Overall, it was concluded that *in vivo* pre-treatment with GPE manifest cardioprotection effect in rats with induced isoproterenol myocardial injury by oxidative stress reduction. The interesting thing within this study is represented by the fact that even if GPE, obtained from the fermented GP, had better *in vitro* antioxidant capacity, thus promising *in vivo* expectations, the GPE obtained from fresh GP with lower *in vitro* antioxidant capacity had better *in vivo* results. These results underline again that the profile of phenolic compounds is paying an important role in inducing cardioprotective effect through antioxidant processes modulation.

That is why, many studies are evaluating the cardioprotective effect of the major phenolic compounds identified in grapes or wine (including RESV), intending to identify the compounds responsible for the pharmacological effects. Thus, the antioxidant capacity of RESV to protect against myocardial ischemic damage or ischemia-reperfusion injury concerning oxidative stress was also studied.

The cardioprotection effect of RESV after ischemia/reperfusion injury through the antioxidant enzymes modulation was studied in rats hearts. Male rats were divided into two groups. One group received an intraperitoneal injection with 25 mg/ kg of RESV/day while the other received an intraperitoneal injection with 10% ethanol/day for 7 days. After the last injection, surgery was performed on rats and, their hearts were removed and preserved to perform ischemia/reperfusion model and to assay further examinations. After 1 week of RESV injection, hearts from the experimental group showed increased contractility, SOD activity, and peroxidase levels and decreased MDA, iron levels, and CAT function. Iron is a catalyst for Fendon reaction in which ROS are formed as hydroxyl radicals. Malondialdehyde was determined to assess lipid peroxidation which was significantly decreased in the RESV group. In the case of CAT there was a significant decrease in function of the enzyme but not in the levels of the enzyme (127).

The cardioprotective effects of RESV after myocardial ischemia and reperfusion were also studied on rats, with no other pre-conditions imposed (128). The rats were divided into two groups (experimental and control). The experimental group received a dose of 10 μM of RESV. The rats suffered cardiac surgery, where their hearts were removed and after that, the hearts were subjected to ischemia/reperfusion lesion (128). For cardiac lesion, the levels of lactate dehydrogenase (LDH), creatine kinase-MB (CK-MB), and troponin I was measured. For oxidative stress, nitrite and MDA levels, CAT, glutathione peroxidase (GSH-Px), and SOD were determined. In the RESV group, there was a significant decrease in levels of LDH, CK-MB, and troponin I (128). Resveratrol showed antioxidant effect by increasing antioxidant enzymes activity (SOD, CAT, GSH-Px) compared to the control group. Lipid peroxidation measured through MDA showed a decrease when compared to the control group. Nitrite levels (a metabolite of nitric oxide) showed also a significant increase when was compared with the control group (128).

Resveratrol showed antioxidant effects on cardiomyocytes subjected to oxidative stress as observed on H9c2 cells (embryonic rat heart cells) (129). Cells were divided into the exposed group, subjected to ischemia/reperfusion lesion, and the control group. In the first set of experiments, cells were pre-treated with 50 μM RESV before ischemia exposure (129). In the second set, cells were transfected with negative control small interfering RNA (NC siRNA) and protein deglycase Dj-1 small interfering RNA (Dj-1 siRNA down-regulation of Dj-1 expression) followed by a dose of 50 μM RESV and exposure to ischemia. In the third set, cells were treated with RESV and after that with 50 μM Sitrinol (SIRT1 inhibitor) and ischemic exposure (129). Suppression of apoptosis and oxidative stress induced by RESV were measured through cell viability, Dj-1, silent information regulator 1 (Sirt1), and tumor protein P53 (p53) expression. Pre-treatment with RESV showed an increased expression of Dj-1 when cells were transfected with NC siRNA compared with cells transfected with DJ-1 siRNA. After the SIRT1 expression was determined, it was found a significant increase in the NC siRNA group pre-treated with RESV. In the group with Dj-1 siRNA, the effect of RESV on SIRT1 expression was reduced. In addition, the positive effect of RESV decreased, when cells received Sitrinol. The link between Dj-1 expression induced by RESV and increased binding of SIRT1 to Dj-1 was assessed. Resveratrol pre-treatment showed increased Dj-1 expression in transfected NC siRNA cells, and also increased binding between SIRT1 and Dj-1. It can be concluded that increased Dj-1 expression by RESV also increased SIRT1 expression. p53 activity was also examined. In the group NC siRNA and pre-treated with RESV, the activity of p53 was significantly suppressed. The group of cells that received Sitrinol showed increased activity of p53. Apoptosis was significantly lower in the group who received pre-treatment with resveratrol with the same tendency toward group transfected with Dj-1 siRNA or SItrinol who showed an increase in apoptotic cells. Dj-1 is a protein with a role in the suppression of oxidative stress and it was discovered that Dj-1 bounds and activates SIRT1 (anti-apoptotic effect) which further suppresses the activity of p53 (pro-apoptotic protein).

Prenatal hypoxia can lead to cardiovascular and metabolic complications in later life (130). Pregnant rats were subjected to hypoxia/normoxia protocol. In the hypoxia protocol, prenatal hypoxia was induced. Later on, the offsprings were randomly divided into 4 groups: normoxia–high fat (HF) diet group, normoxia-HF + RESV group, pre-natal hypoxia-HF group, pre-natal hypoxia-HF + RESV group. After 9 weeks of a high-fat diet, their hearts were isolated through cardiac surgery and subjected to ischemia/reperfusion lesion (*ex vivo*). In terms of heart function *in vivo*, there were no significant changes to influence the global activity of the heart. In *ex vivo*, after induced ischemia/reperfusion lesion, there was a significant decrease of heart recovery from an ischemic event in pre-natal hypoxia group with even significant differences between sexes (90% in male pre-natal hypoxia rats > 50% female pre-natal hypoxia rats) (130). Resveratrol increased heart recovery in males and females rats exposed to pre-natal hypoxia and a high-fat diet. Oxidative stress was significantly higher in pre-natal hypoxia compared to the normoxia group and treatment with RESV showed a significant decrease in oxidative stress who were subjected to the effects of pre-natal hypoxia and a high-fat diet. Summed up, the RESV showed a significant cardioprotective effect.

Even though there are multiple studies suggesting polyphenols effect on the modulation of oxidative stress and other important pathophysiological changes in CAD like lipid metabolism and prevention of atheromatous plaque formation through their antioxidant activities much evidence is still needed especially for the *in vivo* studies. Referring to the antioxidant /pro-oxidant balance of polyphenols action in CAD, there are no literature studies to elucidate both aspects at the same time. Moreover, the existing studies are focused only on the antioxidant effect. Future studies should focus on the antioxidant/ pro-oxidant approach to have a comprehensive view of their action. Also, given the ability of these compounds in reducing important risk factors in CAD and CVD, the use of the grapevine and wine by-products should be indicated as adjunctive therapies only after complex studies covering their safety profile, pharmacokinetics and pharmacodynamics studies, toxicity, and drug interactions. Also, the problem related to the confidence in the use of these products could be solved if the regulations applied to drugs could be applied to these types of natural supplements or nutraceuticals.

## Conclusions

Limited data are available concerning the effect of grapevine by-products toward modulating the antioxidant/pro-oxidant effect *in vitro* or *in vivo* ischemic heart disease conditions. Most of the literature data available and that can offer information in this regard is referring to grapes and wine products or some specific compounds, including RESV, specific for these products. Also, their influence on oxidative stress is mainly discussed concerning their effects on several cardiovascular factors, simulated under various experimental settings. More homogenous experimental studies are needed so that an accurate conclusion could be drawn. The antioxidant/pro-oxidant balance is multifactorial depended, especially dose and time, therefore it is an extremely sensitive process that can be easily shifted. Therefore, the fine line between doses that induce beneficial or undesirable effects should always be investigated. Also, the antioxidant/pro-oxidant effect should be approached globally, a correlation between the plasmatic and the tissues levels in both normal physiological or pathological conditions could offer insight in finding the optimal dose and time for a balanced antioxidant/pro-oxidant effect. Once established, the recommendation of grapevine by-products administration as a preventive measure could be done. Knowing that endothelial dysfunction is a condition that proceed clinically manifested IHD and that the mentioned grapevine and wine by-products, especially grape pomace, grape seed, and resveratrol, in the correct amount, have a positive impact on maintaining the redox endothelial balance as well on the atherosclerosis development we can only think that their preventive administration in persons with high cardiovascular risks will be beneficial.

## Author Contributions

VSC and RMP: conceptualization and writing—original draft preparation. ICB, LLT, MLI, and CMV: methodology. LLT, MLI, ADB, and ICB: validation and visualization. VSC, ŞOM, DCM, and RMP: formal analysis and investigation. LLT and MLI: resources. VSC, CMV, ADB, and RMP: data curation. VSC, ICB, ŞOM, DCM, and RMP: writing—review and editing. RMP: supervision. VSC: project administration. LLT: funding acquisition. All authors have read and agreed to the published version of the manuscript.

## Funding

This work was supported by the Romanian Ministry of Agriculture and Rural Development, Grant Number ADER 7.5.3.

## Conflict of Interest

The authors declare that the research was conducted in the absence of any commercial or financial relationships that could be construed as a potential conflict of interest.

## Publisher's Note

All claims expressed in this article are solely those of the authors and do not necessarily represent those of their affiliated organizations, or those of the publisher, the editors and the reviewers. Any product that may be evaluated in this article, or claim that may be made by its manufacturer, is not guaranteed or endorsed by the publisher.
